# The Additive Effects of Transcranial Direct Current Stimulation Combined With Exercise‐Based Rehabilitation Interventions on Balance, Ankle Kinematics, and Muscle Activity in Individuals With Ankle Instability: A Systematic Review

**DOI:** 10.1002/hsr2.72833

**Published:** 2026-07-20

**Authors:** Alireza Bayati, Razieh Yousefian Molla

**Affiliations:** ^1^ Department of Physical Education and Sport Sciences, CT.C. Islamic Azad University Tehran Iran

**Keywords:** ankle instability, ankle sprain, balance, electromyography, exercise, kinematics, transcranial direct current stimulation

## Abstract

**Background/Aim:**

Transcranial direct current stimulation (tDCS) has been explored as a neuromodulatory technique to enhance sensorimotor function and rehabilitation outcomes in individuals with musculoskeletal disorders, including ankle instability. This study aimed to systematically synthesize the existing evidence on the additive effects of tDCS combined with exercise‐based rehabilitation interventions on balance, ankle kinematics, and muscle activity in individuals with ankle instability.

**Methods:**

The databases of PubMed, Scopus, Cochrane Library, ProQuest, Web of Science, and Science Direct were searched up to October 2025. Eligible studies were randomized controlled trials evaluating the impacts of tDCS on individuals with ankle instability and reporting at least one outcome related to balance, ankle kinematics, or electromyographic activity. Methodological quality was appraised using a modified Downs and Black checklist.

**Results:**

Nine randomized controlled trials (*n* = 298) were included. Overall methodological quality ranged from fair to good, with six studies rated as good and three as fair. Most studies evaluated chronic ankle instability and compared tDCS combined with exercise to exercise alone. Across trials, some studies demonstrated greater improvements in balance and ankle kinematic outcomes in the tDCS + exercise groups, consistent with a potential additive effect, whereas other studies found no between‐group differences. Effects on muscle activation were inconsistent, with only one study showing sustained increases in activation with tDCS + exercise‐based interventions.

**Conclusion:**

When used as an adjunct to exercise‐based rehabilitation interventions, tDCS shows preliminary and context‐dependent additive effects on balance and ankle kinematics in individuals with ankle instability, while evidence for effects on muscle activation remains inconsistent. Current data are insufficient to support routine clinical use due to methodological heterogeneity and inconsistent evidence across studies, and further methodologically standardized trials are required.

## 1 Introduction

Ankle sprains are among the most common musculoskeletal injuries, particularly in physically active individuals and athletes [1, 2]. Approximately 20%–40% of individuals who sustain a lateral ankle sprain may subsequently develop chronic ankle instability (CAI) [3], characterized by recurrent sprains, persistent symptoms, neuromuscular deficits, proprioceptive impairments, and altered postural control [3, 4]. In addition to chronic symptoms, sensorimotor and functional deficits may also be present during the acute phase following ankle sprain. Across both acute and chronic phases, ankle instability can negatively affect physical performance, daily and sports activities, and quality of life, while increasing the risk of recurrent injury and early joint degeneration [2, 4]. Because many individuals continue to experience residual symptoms despite conventional rehabilitation, there is growing interest in adjunctive therapeutic approaches that may further enhance sensorimotor recovery and functional outcomes [1, 2].

Conventional rehabilitation programs for ankle instability primarily emphasize exercise‐based interventions targeting proprioception, neuromuscular control, balance, and muscle strengthening [5, 6]. These rehabilitation approaches are intended to improve postural stability, sensorimotor function, and dynamic joint control during functional activities. Although such interventions can enhance functional performance and reduce symptoms in many individuals, persistent impairments in balance, proprioception, and perceived instability are still frequently reported following rehabilitation [7, 8, 9]. Because many individuals continue to experience residual symptoms despite conventional rehabilitation, there is growing interest in adjunctive therapeutic approaches that may further enhance sensorimotor recovery and functional outcomes. To optimize rehabilitation effects, non‐invasive brain stimulation techniques such as transcranial direct current stimulation (tDCS) have recently been explored as potential adjunctive interventions in musculoskeletal rehabilitation settings [10, 11].

tDCS is a non‐invasive neuromodulation technique that modulates cortical excitability by delivering low‐intensity electrical currents to targeted cortical regions, most commonly the primary motor cortex (M1) or the supplementary motor area (SMA). Evidence from neurological and musculoskeletal populations suggests that tDCS can influence motor learning, sensory processing, and postural control, although the magnitude and consistency of these effects vary across studies due to differences in stimulation protocols and individual responsiveness [12, 13, 14, 15]. Individuals with CAI frequently demonstrate persistent deficits in proprioception, neuromuscular coordination, and postural stability, reflecting both peripheral and central alterations in sensorimotor function [16]. These neurophysiological changes have led researchers to explore whether neuromodulatory techniques such as tDCS may augment traditional exercise‐based rehabilitation approaches aimed at improving balance and functional ankle control. Some randomized controlled trials (RCTs) have applied tDCS, either alone or combined with exercise‐based interventions, in individuals with ankle instability and reported mixed results across measures of balance, ankle joint mechanics, and muscle activation [17, 18]. This variability underscores the need for a comprehensive synthesis of available evidence to better understand the potential role of tDCS in the rehabilitation of ankle instability.

Although several reviews have examined the application of tDCS in sports performance [14] and neurorehabilitation settings, including populations with stroke, multiple sclerosis, and Parkinson's disease [15, 19, 20], evidence regarding its role in ankle instability rehabilitation remains limited. In addition, a recent systematic review and meta‐analysis evaluated the effects of exercise therapy combined with tDCS on balance outcomes in individuals with CAI [21]. However, previous reviews have primarily focused on balance‐related outcomes and have not comprehensively synthesized evidence related to ankle kinematics and muscle activation. Therefore, the purpose of the present systematic review was to examine the additive effects of combining tDCS with exercise‐based rehabilitation interventions on balance, ankle kinematics, and muscle activity in individuals with ankle instability.

## 2 Methods

### 2.1 Design

The present systematic review adhered to the PRISMA guidelines for transparent and standardized reporting. The review protocol was prospectively registered in the PROSPERO database (registration ID: CRD420251108595).

### 2.2 Literature Search

A comprehensive literature search was performed across multiple electronic databases, including PubMed, Scopus, Cochrane Library, ProQuest, Science Direct, and Web of Science, covering studies from inception to October 2025 (Appendix S1). Gray literature sources, including theses and conference papers indexed within ProQuest, Scopus, and ISI Web of Science, were also screened as part of the search process. The search strategy was formulated based on the PICO framework (Population, Intervention, Comparison, and Outcome), and a combination of relevant keywords was used to retrieve eligible studies including: (“non‐invasive brain stimulation” OR “Transcranial Electrical Stimulation” OR “Transcranial direct current stimulation” OR “Transcranial current stimulation” OR “neuromodulation” OR “tDCS”) AND (“ankle sprain” OR “ankle instability” OR “ankle inversion” OR “syndesmotic injury” OR “syndesmotic injuries” “ankle injury” OR “ankle injuries”) AND (“balance” OR “equilibrium” OR “postural sway” OR “postural stability” OR “center of pressure” OR “kinematic” OR “walking” OR “gait” OR “standing” OR “muscle activity” OR “muscle activation” OR “electromyography”). Two review authors (A. B. and R. Y. M.) independently provided the search process and screened articles, and any disagreements in their assessments were resolved through negotiation.

### 2.3 Inclusion and Exclusion Criteria

Studies were included if they met the following criteria [1]: RCTs examining the effects of tDCS in individuals with acute or CAI [2]; assessment of at least one relevant outcome—balance, ankle kinematics, or muscle activity; and [3] use of sham stimulation as the control condition. Exercise‐based rehabilitation interventions were broadly defined to include strengthening, balance, sensorimotor, mobility, eccentric, and movement‐based therapeutic interventions commonly used in ankle instability rehabilitation. Exclusion criteria comprised [1]: study protocols, case reports, conference abstracts, reviews, or meta‐analyses [2]; animal‐based research; and [3] insufficient data for assessing risk of bias.

### 2.4 Data Extraction

A researcher (A. B.) performed the screening and data extraction in the following way: authors and publication year, trial design, participants, study groups, intervention duration and intensity of tDCS, exercise‐based rehabilitation interventions, outcome measures, follow‐up assessments, and conclusion. Detailed characteristics of the included studies are presented in Table 2.

### 2.5 Quality Assessment

Study quality and potential bias were appraised using a modified version of the Downs and Black checklist [22], as previously adapted in similar systematic reviews on ankle instability [23, 24]. This instrument assesses five domains of bias: (a) reporting, (b) external validity, (c) internal validity (bias), (d) internal validity (confounding/selection bias), and (e) statistical power. Each domain was rated as “yes” (score = 1), “no” (score = 0), or “unclear” (score = 0). Two reviewers (A. B. and R. Y. M.) conducted independent evaluations of all items, and any discrepancies in scoring were resolved through discussion. The original 27‐item checklist was modified by excluding items 5, 16, and 25, which were considered insufficiently applicable or difficult to interpret consistently across the included RCTs (Table 1). Specifically, the removed items related to the description and adjustment of confounding factors and the identification of unplanned post hoc analyses, which were either uniformly unreported or difficult to assess reliably within the included studies. Overall quality scores of the 24‐item checklist were classified into three categories: good (≥ 75% of the maximum possible score), fair (50%–74%), and low (< 50%), based on predefined cut‐off values. consistent with previous systematic review protocols and empirical studies [30, 31]. Two reviewers (A. B. and R. Y. M.) independently rated each study, and discrepancies were resolved through discussion.

**Table 1:** Downs and Black quality checklist.

				Report							External validity			Internal validity – Bias						Internal validity – Confounding					Power	Total	Quality status
	Quality items	Q1	Q2	Q3	Q4	Q6	Q7	Q8	Q9	Q10	Q11	Q12	Q13	Q14	Q15	Q17	Q18	Q19	Q20	Q21	Q22	Q23	Q24	Q26	Q27		
		Hypothesis/aim	Main outcomes in method/introduction	Inclusion/exclusion criteria	Description of interventions	Main findings	Random variability	Adverse events	Lost to follow up	Actual probability values	Representative of the entire population	Representative (subjects who were prepared) of the entire population	Representative of the treatment	Blind study subjects	Blind those measuring	Different lengths of follow‐up	Statistical tests appropriated	Compliance with the intervention	Outcome measure used accurate	Patients in different groups recruited from the same population	Same period of time	Random allocation	Concealed allocation	Losses of patients to follow up	Estimate of statistical power		
1	Beyraghi et al. [25]	1	1	1	1	1	1	0	1	1	0	0	0	1	0	1	1	1	1	1	0	1	1	1	1	18	Good
2	Beyraghi et al. [26]	1	1	1	1	1	1	1	1	1	0	0	0	1	0	1	1	1	1	1	0	1	1	1	1	19	Good
3	Kim et al. [12]	1	1	1	1	1	1	1	1	1	0	0	0	1	0	1	1	1	1	1	1	1	0	1	1	19	Good
4	Needle et al. [27]	1	1	1	1	1	1	0	1	1	0	0	0	1	1	1	1	1	1	1	0	1	0	1	1	18	Good
5	Gao et al. [18]	1	1	1	1	1	1	0	1	1	0	0	0	1	0	1	1	0	1	1	0	1	0	1	1	16	Fair
6	Huang et al. [28]	1	1	1	1	1	1	0	1	1	0	0	0	1	0	1	1	1	1	1	0	1	1	1	1	18	Good
7	Chaturvedi et al. [17]	1	1	1	1	1	1	0	1	1	0	0	0	1	0	1	1	1	1	1	0	1	0	1	0	16	Fair
8	Ma et al. [11]	1	1	1	1	1	1	1	1	1	0	0	0	1	1	1	1	1	1	0	0	1	0	1	1	18	Good
9	Bruce et al. [29]	1	1	1	1	1	1	0	1	1	0	0		1	0	1	1	1	1	0	0	1	0	1	1	16	Fair

## 3 Results

### 3.1 Search Results

**Figure 1:**
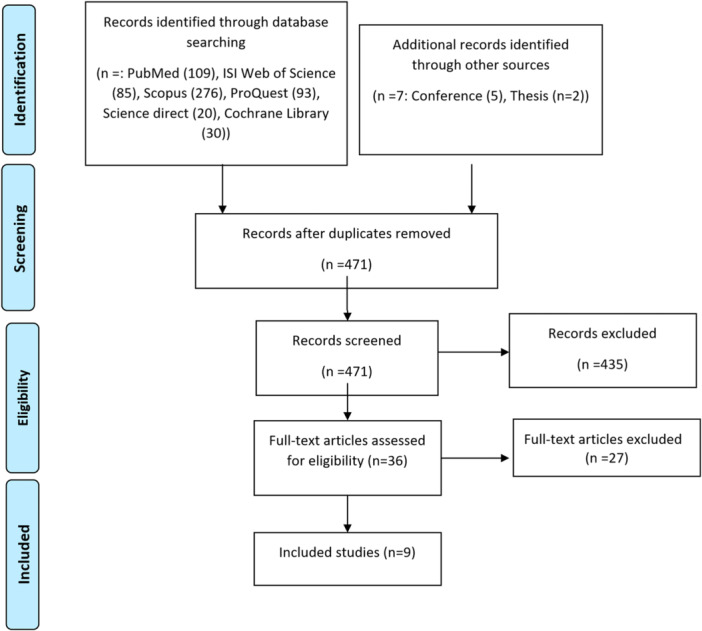
Flow diagram of the study selection based on PRISMA.

The inclusion and exclusion procedure is illustrated in Figure 1. Following the removal of duplicates, 471 records remained, of which 435 were excluded based on title and abstract screening. A total of 36 articles proceeded to full‐text evaluation, after which 1 conference abstract and 1 unpublished study protocol were excluded. Ultimately, 9 studies published between 2020 and 2025 investigating the impact of tDCS on balance, ankle kinematics, and muscle activity in individuals with ankle instability met the eligibility criteria and were included in this systematic review. No language restrictions were considered when selecting studies; however, all eligible studies retrieved through the search were published in English.

A meta‐analysis (quantitative synthesis) could not be carried out due to variations in the outcome measures. Therefore, the results are presented narratively.

### 3.2 Quality Assessment

Table 1 presents the results of the modified Downs and Black checklist. Using the predefined thresholds on previous literature [30, 31], six studies received good scores [11, 12, 25, 26, 27, 28], and three studies received fair scores [17, 18, 29]. Two RCT studies implemented a double‐blinded trial [11, 27], and the other seven studies were single‐blinded trial on participants [12, 17, 18, 25, 26, 28, 29].

### 3.3 Participant Characteristics

The final evaluation encompassed 9 studies involving a total of 298 individuals with ankle instability. All injuries included in these studies occurred during competitive or recreational sports activities. The effect of tDCS was evaluated during the chronic phase of ankle instability for eight studies, except one study for the acute phase [17]. Because the primary objective of the review was to evaluate the effects of tDCS across ankle instability populations and related sensorimotor outcomes, the acute‐phase study was retained and interpreted separately where appropriate. For the studies in the chronic phase, all participants had a history of the first ankle instability more than 1 year ago. For that single study in the acute phase [17], participants had acute ankle instability with symptom duration of less than 2 months following ankle sprain.

### 3.4 Intervention Characteristics (tDCS and Exercise‐Based Rehabilitation Interventions)

Detailed study‐specific tDCS protocols and intervention characteristics are summarized in Table 2. All included trials used a parallel‐group randomized controlled design. Eight of the nine studies incorporated a sham (placebo) stimulation condition as the control. Among these eight trials, seven implemented exercise‐based rehabilitation interventions in both the real‐tDCS and sham groups [11, 18, 25, 26, 27, 28, 29], whereas one study—Chaturvedi et al. [17]—applied real or sham tDCS without any accompanying exercise therapy program. The remaining study, Kim et al. [12], compared real tDCS combined with joint‐biased rehabilitation to active joint mobilization alone and did not include a sham condition.

**Table 2:** Main characteristics and outcomes of the reviewed articles.

References	Study design	Participants	Study groups	Intervention duration and intensity of tDCS	Exercise‐based rehabilitation interventions	Outcome measures	Follow‐up assessments	Conclusion
Beyraghi et al. [25]	Single blind RCT (participants)	32 subjects (23 male, 9 female) with CAI, first acute ankle sprain > 1 year ago, CAIT ≤ 24, age: 18–40 years	– Experimental group (*n* = 16): Anodal tDCS + balance training – Control group (*n* = 16): Sham tDCS + balance training	– 12 sessions, 3 sessions per week, and 60 min per session – 1.5 mA intensity over SMA	Single‐limb stance, limb stance with ball kicking, single‐limb hop to stabilization, and hop to stabilization and reach	Dynamic balance: COP‐related parameters (displacement and velocity) during phases of GI (force plate)	– Baseline – Post‐training	Improvements were observed in both groups during the anticipatory and execution phases of GI (*p* < 0.01); however, the absence of significant between‐group differences (*p* ˃ 0.05) suggests that anodal tDCS over the SMA may not provide additional benefit compared to sham stimulation.
Beyraghi et al. [26]	Single blind RCT (participants)	30 subjects (21 male, 9 female) with CAI, first acute ankle sprain > 1 year ago, CAIT ≤ 24, age: 18–40 years	– Experimental group (*n* = 15): Anodal tDCS + balance training – Control group (*n* = 15): Sham tDCS + balance training	– 12 sessions, 3 sessions per week, and 60 min per session – 1.5 mA intensity over SMA	Single‐leg stance, single‐leg hopping, and hopping	Dynamic balance: APA as COP trajectory during GI (force plate)	– Baseline – After completing the 4‐week intervention	The findings showed no significant interaction effect between group and time for dynamic balance (*p* ˃ 0.05), indicating that real tDCS did not offer any additional advantage over sham stimulation.
Kim et al. [12]	Single blind RCT (participants)	36 subjects (23 male, 13 female) with CAI, A history of at least two ankle sprains on the same side within 2 years, CAIT ≤ 24, age: 20–29 years	– tDCS with joint mobilization (*n* = 12) – active joint mobilization (*n* = 12) – tDCS with active joint mobilization (*n* = 12)	– 12 sessions, three times per week, for 15 min per session, over 4 weeks – 2 mA intensity over the primary motor cortex (M1)	Joint mobilization after beginning tDCS	– Dynamic balance (Y‐balance test) – Static balance (force plate)	– Baseline – After completing the 4‐week intervention	Although all intervention groups exhibited significant gains in both static and dynamic balance (*p* < 0.05), the group receiving tDCS combined with active joint mobilization consistently showed greater effect sizes across balance‐related measures.
Needle et al. [27]	Double blind RCT	44 subjects (15 male, 29 female) with CAI, first acute ankle sprain > 1 year ago, IdFAI > 10, age: 18–30 years	– Experimental group (*n* = 14): Anodal tDCS on motor cortex + exercises – Experimental group (*n* = 11): Anodal tDCS on frontal cortex + exercises – Control group (*n* = 12): Sham tDCS + exercises	– 18–20 min on eight training sessions during 4 weeks – 1.5 mA intensity over the primary motor or frontal cortex	Bosu ball training barefoot: Using a progressive training program	– Dynamic balance (COP on force plate) – Muscle activation (electromyography)	– Baseline: Week‐2 (training mid‐point) – Week‐4 (training completion) – Week‐ 6 (retention)	Muscle activation variables did not differ significantly between groups or over time (*p* > 0.05). However, all groups demonstrated significant improvements in dynamic postural stability indices from baseline (*p* < 0.05).
Gao et al. [18]	Single blind RCT (participants)	40 subjects (23 male, 9 female) with CAI, experiencing at least one severe ankle sprain within the past year, CAIT ≤ 24, age: 18–40 years	– Experimental group (*n* = 20): Anodal tDCS + Bosu ball training – Control group (*n* = 20): Sham tDCS + Bosu ball training	6 weeks with three 20‐min sessions per week, 2 mA intensity over the primary motor cortex	Bosu ball training barefoot: Using a progressive training program	Ankle kinematic data during side‐cutting (3D infrared motion capture system)	– Baseline – After (Week 7) the intervention	Significant group‐by‐intervention interactions were found for ankle maximum inversion (*p* = 0.018) and internal rotation angles (*p* = 0.023). Both groups showed reductions in these angles from Week 0 to Week 7, with more pronounced improvements observed in the tDCS combined with Bosu ball training group compared to Bosu ball training alone.
Huang et al. [28]	Single blind RCT (participants)	40 subjects (23 male, 9 female) with CAI, first acute ankle sprain > 1 year ago, CAIT ≤ 24, age: 18–40 years	– Experimental group (*n* = 20): Anodal tDCS + Bosu ball training – Control group (*n* = 20): Sham tDCS + Bosu ball training	6 weeks with three 20‐min sessions per week, 2 mA intensity over the primary motor cortex	Bosu ball training barefoot: Using a progressive training program	– Kinematic data of ankle (3D infrared motion capture system)	– Baseline – After (Week 7) the intervention	tDCS + Bosu training has better effects in reducing peak ankle inversion angular velocity, plantarflexion angle at the moment of peak ankle inversion and advancing time to peak ankle inversion than Bosu training only (*p* < 0.05). Bosu ball training, as well as tDCS + Bosu ball training, reduced the peak ankle inversion angle, and tDCS + Bosu ball training did not show an extra effect.
Chaturvedi et al. [17]	Single blind RCT (participants)	20 players (14 male, 6 female) with acute ankle sprain, duration less than 2 months of ankle sprain, age: 16–30 years	– Experimental group (*n* = 10): Anodal tDCS – Control group (*n* = 10): Sham tDCS	– 20 min once daily for 5 consecutive days – 2 mA intensity over the primary motor cortex	—	– Balance (Y‐balance test)	– Baseline – After completing the intervention	The application of tDCS is ineffective for improving balance in players with ankle sprains (*p* = 0.502).
Ma et al. [11]	Double blind pilot RCT	30 subjects (15 male, 15 female) with CAI, first acute ankle sprain > 1 year ago, CAIT ≤ 24, age: 18–35 years	– Experimental group (*n* = 15): High‐definition tDCS + short‐foot exercise – Control group (*n* = 15): Sham tDCS + short‐foot exercise	Four‐week intervention, three intervention sessions per week with a break of 24–48 h between each session, 2 mA intensity over the primary motor cortex	Short‐foot exercise training on proprioception and dynamic balance performance	– Proprioception (Joint position reproduction test) – Dynamic balance (Y‐balance test) – Dynamic balance (Sensory organization test) – Proprioception (AMEDA)	– Baseline – After the first, fourth, eighth, and twelfth sessions of intervention – One and two weeks following the last session of intervention	The group receiving high‐definition tDCS combined with short‐foot exercises showed significantly greater improvements in all balance assessments at various time points compared to the control group (*p* ˃ 0.05).
Bruce et al. [29]	Single blind RCT (participants)	26 subjects (9 male, 17 female) with CAI, first acute ankle sprain > 1 year ago, IdFAI > 10, age: 18–40 years	– Experimental group (*n* = 13): Anodal tDCS + exercises – Control group (*n* = 13): Sham tDCS + exercises	– 18 min duration of eccentric testing, 10 training sessions, 2–3 sessions per week – 1.5 mA intensity over the primary motor cortex	Eccentric ankle exercises on an isokinetic dynamometer	– Dynamic balance (COP on force plate) – Muscle activation (electromyography)	– Baseline: Week‐2 (training mid‐point) – Week‐4 (training completion) – Week‐ 6 (retention)	tDCS with eccentric training increased enhances dynamic postural stability (*p* = 0.034) following 4 weeks of training in patients with CAI.

Abbreviations: AMEDA, Active Movement Extent Discrimination Apparatus; APA, anticipatory postural adjustments; CAI, chronic ankle instability; CAIT, Cumberland Ankle Instability Tool; COP, center of pressure; GI, gait initiation; IdFAI, Identification of Functional Ankle Instability; SMA, supplementary motor area; tDCS, transcranial direct current stimulation.

Regarding stimulation targets, most studies applied anodal tDCS to motor‐related cortical areas, although electrode montages varied across trials. Seven studies stimulated the M1 using an anode positioned over Cz or adjacent motor regions, with return (cathodal/return) electrodes placed at locations such as Fz, Pz, C3, or C4, depending on the specific protocol [11, 12, 17, 18, 27, 28, 29]. Two studies targeted the SMA using a midline frontal anode placement [25, 26]. One study additionally applied a dorsolateral prefrontal cortex (DLPFC) montage, with the anode positioned at F3 or F4 according to the 10–20 EEG system and the return electrode on the contralateral homologous site [27]. Because montage configurations differed across studies, detailed electrode placements for each trial are provided in Table 2.

Current intensities of tDCS ranged from 1.5 to 2 mA, and session durations varied between 15 and 60 min, with 20 min being the most common. Total intervention duration ranged from 4 to 18 sessions (most frequently 12 sessions).

The rehabilitation interventions combined with tDCS varied across studies and included balance training, eccentric ankle exercises, Bosu ball training, short‐foot exercises, agility and dual‐task training, and joint mobilization approaches. In seven of the nine studies, tDCS (active or sham) was administered alongside balance‐ or strength‐based exercise programs, including Bosu ball training [18, 28], short‐foot exercise [11], single‐limb stance and hop‐based tasks [25, 26], obstacle walking and agility training [27], and eccentric evertor strengthening on an isokinetic dynamometer [29]. One study also incorporated joint mobilization–based rehabilitation approaches, including active mobilization techniques of the ankle joint, rather than conventional balance or strengthening exercises [12]. Chaturvedi et al. [17] was the only study conducted during the acute phase of ankle instability and did not include any exercise or balance‐training component. All sham protocols used electrode placements identical to those in active tDCS conditions but without delivering sustained current.

### 3.5 Study Outcomes

Balance assessment was the primary outcome in seven studies and included both dynamic and static balance measures. Dynamic balance was assessed by measuring joint position reproduction test [11], Y‐balance test [12, 17], sensory organization test [11], hop to stabilization test [11, 27, 29], the center of pressure (COP)–related parameters (displacement and velocity in anterior‐posterior and medial‐lateral directions, and anticipatory postural adjustments duration) when gait initiation on the force plate [25, 26], and or dynamic postural stability indices including anteroposterior, mediolateral, and vertical components during reactive hop task [27]. Static balance was evaluated using a force plate that monitored COP displacement while participants stood on one leg, specifically on the unstable limb [12]. Proprioception as a balance‐related outcome was measured by the joint position reproduction test and Active Movement Extent Discrimination Apparatus (AMEDA) in one study [11].

Two studies assessed ankle kinematics using a 3D infrared motion capture system during side‐cutting or the drop‐landing test [18, 28]. Two studies assessed electromyography activity of the peroneus longus, tibialis anterior, and soleus muscles during functional motor tasks, including reactive hop stabilization and eccentric strengthening [27, 29].

### 3.6 Main Findings

#### 3.6.1 Effect of tDCS on Balance Outcomes

Two investigations explored the effects of tDCS targeting the SMA on COP parameters during gait initiation in individuals with CAI. The results showed that both the real tDCS + balance training and sham tDCS + balance training groups demonstrated significant post‐treatment improvements in Y‐balance performance, COP displacement, and anticipatory postural adjustments. For example, Beyraghi et al. reported significant improvements in dynamic balance directions (*p* ≤ 0.010, *η* ^2^ = 0.204–0.350) and perceived ankle instability scores (*p* < 0.001, *η* ^2^ = 0.391) in both groups, without significant group‐by‐time interaction effects [26]. Similarly, anticipatory and locomotor COP parameters improved over time in both groups (*p* = 0.02 to *p* < 0.01), with no significant between‐group differences (*p* > 0.05) [25]. In another study by Needle et al., the findings indicated that across all groups (motor tDCS, frontal tDCS, and sham tDCS), participants displayed improvements in dynamic postural stability indices, including anteroposterior, mediolateral, and vertical components, during lateral hops following the 4‐week intervention. However, no significant group or group‐by‐time interaction effects were noted for any dynamic postural control variables [27]. In contrast, Bruce et al. concluded that in the real tDCS combined with exercise group, anteroposterior postural stability indices showed a reduction from baseline and Week 2 to Week 6, reflecting enhanced balance performance. For the control group, no significant changes were detected in the sham tDCS plus exercise group [29].

Ma et al. investigated the impact of high‐definition tDCS combined with short‐foot exercise on dynamic balance and proprioception. Their findings revealed significant time × group interactions for joint position reproduction at 15° inversion (*p* = 0.029, *η* ^2^ = 0.102) and composite Y‐balance reach distance (*p* = 0.044, *η* ^2^ = 0.096). Post hoc analyses further demonstrated sustained improvements in dynamic balance from Week‐4 through Week‐6 in the high‐definition tDCS group compared with sham stimulation [11].

Kim et al. explored the effects of combining tDCS with joint mobilization techniques (as exercise‐based rehabilitation) in individuals with CAI. Participants were assigned to one of three groups: (1) tDCS plus joint mobilization, (2) tDCS plus active joint mobilization, and (3) active joint mobilization alone. While all groups experienced significant improvements over time in both static and dynamic balance outcomes (*p* < 0.05), no significant between‐group interaction effects were observed (*p* > 0.05). However, the group receiving tDCS combined with active joint mobilization demonstrated relatively larger within‐group effect sizes for dynamic balance (post‐intervention: *d* = 0.929; follow‐up: *d* = 1.787), ankle instability (post‐intervention: *d* = 2.230), and dorsiflexion ROM (follow‐up: *d* = 3.300), despite the absence of significant between‐group interaction effects [12].

Regarding the acute phase of ankle instability, only one article evaluated the impact of tDCS on balance. Chaturvedi et al. reported no statistically significant difference in dynamic balance, as measured by the Y‐Balance Test (*p* = 0.502), when comparing the real tDCS group to the sham tDCS group in athletes with acute ankle instability [17].

#### 3.6.2 Effect of tDCS on Ankle Kinematics

Two studies investigated the influence of tDCS on ankle kinematics [18, 28] using a 3D infrared motion capture system. In both studies, participants performed Bosu ball training combined with either real or sham tDCS. In the study by Gao et al., significant reductions were observed in maximum ankle inversion (*p* = 0.018) and internal rotation angles (*p* = 0.023) during side‐cutting movements post‐intervention (Week 7) compared to pre‐intervention (Week 0) in both groups. However, these improvements were significantly greater in the group receiving real tDCS along with balance training than in the sham group, with moderate effect sizes observed for maximum ankle inversion angle (*η* ^2^ *p* = 0.162) and internal rotation angle (*η* ^2^ *p* = 0.151), suggesting a reduced risk of injury. Additionally, both groups showed similar directional improvements in jump displacement (*p* < 0.001), push‐off impulse (*p* < 0.001), and take‐off velocity (*p* = 0.002), indicating enhanced overall movement performance. Notably, push‐off impulse demonstrated the largest intervention‐related effect (*η* ^2^ *p* = 0.770) [18]. In another study, Huang et al. found that participants in the real tDCS + Bosu training group experienced greater reductions in peak ankle inversion angular velocity and plantarflexion angle at peak inversion, as well as a faster time to reach peak inversion, compared to the sham group. Significant interaction effects were observed for peak inversion angular velocity (*p* = 0.047, *η* ^2^ *p* = 0.118), time to peak inversion (*p* = 0.030, *η* ^2^ *p* = 0.139), and plantarflexion angle at peak inversion (*p* = 0.014, *η* ^2^ *p* = 0.173). Both groups, however, showed significant decreases in peak ankle inversion angle following the intervention (Week 7) versus baseline (Week 0) (*p* < 0.001) [28].

Collectively, these findings suggest that combining tDCS with Bosu ball training may influence selected ankle kinematic parameters during dynamic tasks such as drop landing and side‐cutting in individuals with CAI. However, because only two studies evaluated ankle kinematics and some outcomes improved similarly in both groups, these findings should be interpreted cautiously [18, 28].

#### 3.6.3 Effect of tDCS on Muscle Activation

Two studies examined the impact of tDCS on muscle activation. Needle et al. compared three groups receiving motor tDCS, frontal tDCS, and sham stimulation. Their results showed no significant main effects of time, group, or group‐by‐time interaction on the average activation levels of the peroneus longus, tibialis anterior, and soleus muscles during a reactive hop task [27]. In contrast, Bruce et al. found that while the sham tDCS combined with eccentric exercise group exhibited improved muscle activity from baseline to Week 2, these gains were not maintained through Weeks 4 and 6. Conversely, the group receiving real tDCS along with exercise demonstrated sustained increases in muscle activation from baseline to Week 6 (*p* = 0.034). In addition, cortical excitability of the peroneus longus improved in the aTDCS group from baseline (36.92 ± 11.53) to Week 6 (32.91 ± 12.33; *p* = 0.024) [29].

## 4 Discussion

Previous reviews have demonstrated the benefits of tDCS in enhancing balance and motor performance in populations with stroke, multiple sclerosis, and Parkinson's disease [15, 19, 20]. Although research on the use of tDCS within musculoskeletal rehabilitation is still emerging, the available evidence provides only limited and preliminary indications that neuromodulatory approaches may be helpful for individuals with joint instability. A recent systematic review and meta‐analysis primarily focused on balance‐related outcomes following combined tDCS and balance‐based exercise interventions in individuals with CAI [21]. While the present review includes substantial overlap in balance‐related studies, it extends the current literature by additionally synthesizing evidence related to ankle kinematics and muscle activation outcomes, which have received considerably less attention. Moreover, the present review adopted a broader rehabilitation perspective by including not only exercise‐based interventions but also mobilization‐based rehabilitation approaches combined with tDCS. In addition, one included study evaluated participants during the acute phase following ankle sprain, whereas previous reviews primarily focused on CAI populations. Overall, the findings suggest that tDCS—particularly when combined with rehabilitation interventions—may offer potential benefits in some studies; however, the evidence remains inconsistent, the magnitude of effects is modest, and several methodological limitations temper the strength of these conclusions. Nevertheless, because evidence for ankle kinematics, muscle activation, and acute‐phase application remains limited and heterogeneous, these findings should currently be interpreted as exploratory.

### 4.1 Effects on Balance

Balance was the most frequently assessed outcome across the included studies, with dynamic and static balance evaluated using a variety of functional and instrumented measures. Because static and dynamic balance involve different postural control demands and sensorimotor mechanisms, direct comparison across studies using different balance paradigms should be interpreted cautiously. Several studies reported positive effects of combining tDCS with balance or proprioceptive training on postural control outcomes. For example, Ma et al. [11] and Kim et al. [12] observed significant improvements in joint position sense and Y‐balance performance when tDCS was combined with short‐foot exercises or joint mobilization, respectively. These findings align with previous work suggesting that anodal tDCS applied to the motor cortex can enhance motor learning and sensorimotor integration in neurologic and musculoskeletal populations [10, 14, 15]. From a neurophysiological perspective, balance control depends on continuous integration of sensory input and motor output across distributed cortical and sensorimotor networks [32]. Stimulation of the M1 may enhance corticospinal excitability and sensorimotor integration, thereby improving postural control and motor adaptation during balance‐related tasks [33]. In contrast, stimulation of other cortical regions, such as the SMA or DLPFC, may influence motor planning, attentional control, or dual‐task processing differently, potentially contributing to variability in balance‐related outcomes across studies [33].

However, not all studies reported superior outcomes in the tDCS groups compared to sham stimulation. Beyraghi et al. [25, 26] and Needle et al. [27] found no significant between‐group differences in dynamic balance indices despite improvements over time in both groups. This suggests that while task‐specific training alone is effective, the additive benefit of tDCS remains uncertain in certain protocols or target areas (e.g., SMA vs. M1).

Differences in exercise modality may partially explain the variability in balance‐related findings across studies. Trials combining tDCS with proprioceptive or sensorimotor‐focused interventions, such as short‐foot exercises, Bosu ball training, or joint mobilization approaches, generally reported more favorable outcomes than studies using reactive hopping or agility‐based protocols. It is possible that interventions emphasizing controlled sensorimotor activation and postural regulation are more responsive to cortical neuromodulation than highly dynamic tasks requiring complex whole‐body coordination [34]. In addition, task specificity may be important, as tDCS‐induced modulation of motor cortical excitability is thought to be most effective when paired with repeated practice of the targeted motor function [34, 35, 36].

Interestingly, the only study conducted during the acute phase of injury [17] did not find any significant effect of tDCS on balance. One possible explanation is that the absence of concurrent balance or proprioceptive exercises may have contributed to the lack of improvement in postural outcomes [17]. This may reflect the timing of the intervention, as cortical plasticity and sensorimotor deficits may be more prominent during the chronic phase of ankle instability, making it a more favorable target for neuromodulation strategies [37].

### 4.2 Effects on Ankle Kinematics

Two studies evaluated the influence of tDCS on ankle joint mechanics during dynamic tasks such as side‐cutting and drop‐landing [18, 28]. In both studies, the combination of tDCS with balance exercises was associated with improvements in several ankle kinematic parameters during dynamic tasks. However, statistically significant between‐group differences were not consistently observed across all variables; some outcomes improved similarly in both the real and sham tDCS groups. These findings may suggest a potential role for tDCS in modulating neuromuscular control strategies associated with safer and more stable movement patterns in individuals with CAI. Ankle kinematics during dynamic tasks such as landing and side‐cutting depend heavily on rapid sensorimotor processing and precise neuromuscular coordination. Anodal stimulation over M1 may facilitate cortical regulation of lower‐limb muscle activation patterns and improve anticipatory motor control, which could contribute to reductions in excessive inversion or altered landing mechanics [38]. Because both kinematic studies [18, 28] primarily targeted sensorimotor‐related cortical regions during dynamic balance training, the observed effects may reflect enhanced integration between cortical motor planning and movement execution [39].

Notably, both kinematic studies paired tDCS with Bosu ball–based balance training during dynamic functional tasks. This consistency in intervention type may partially explain why both studies reported at least some favorable kinematic adaptations. Because these exercises require continuous sensorimotor adjustment and rapid postural corrections, they may provide an optimal behavioral context for enhancing task‐relevant cortical plasticity through tDCS [34, 35].

Improved ankle kinematics, such as reduced inversion and better timing of joint movements, have been associated with lower risk of recurrent sprains and injury [4, 40]. Therefore, the available evidence may indicate a potential role for tDCS in facilitating motor adaptations related to joint stability when paired with targeted rehabilitation interventions. Nevertheless, given the limited number of studies and partially inconsistent findings, further research is required before firm conclusions can be drawn.

### 4.3 Effects on Muscle Activation

Only two studies evaluated muscle activation using surface EMG during functional motor tasks [27, 29]. Bruce et al. assessed activation of the ankle evertor musculature during eccentric strengthening exercises, whereas Needle et al. evaluated lower‐extremity muscle activation patterns during a reactive hop stabilization task. Their findings were inconsistent. Bruce et al. [29] reported sustained increases in ankle stabilizer activation following real tDCS combined with eccentric evertor strengthening, whereas Needle et al. [27] found no differences between M1‐tDCS, DLPFC‐tDCS, and sham during a reactive hop task.

These divergent findings likely arise from substantial methodological differences between the two trials. Bruce et al. [29] applied anodal stimulation over the M1 during a structured eccentric strengthening program targeting the ankle evertors, a task directly related to the muscles being evaluated. In contrast, Needle et al. [27] used two different cortical targets (M1 and DLPFC), administered stimulation during obstacle walking and agility tasks that did not specifically load the muscles monitored via EMG, and assessed activation during a reactive hop task—a demanding movement potentially influenced by whole‐body coordination rather than localized neuromuscular control. In addition, cortical target selection may be important, as stimulation over M1 is more directly linked to modulation of corticospinal output and muscle recruitment [38], whereas stimulation over regions such as the DLPFC may primarily affect cognitive or attentional aspects of motor performance [10]. tDCS may influence muscle activation by enhancing corticospinal excitability [38] and activity‐dependent neuroplasticity [34] within motor cortical networks. Anodal stimulation over M1 has been shown to induce sustained increases in cortical excitability, potentially facilitating voluntary muscle activation during task performance [38]. These effects may be enhanced when tDCS is combined with task‐specific rehabilitation exercises that repeatedly engage the targeted neuromuscular pathways [41].

Additional discrepancies include differences in stimulation dose (1.5 vs. 2 mA), intervention duration (4 weeks vs. 6 weeks), and type of training paired with tDCS (strength‐focused vs. balance/agility‐based). Prior mechanistic literature indicates that tDCS effects on neuromuscular output are task‐ and context‐dependent, and more pronounced when stimulation is paired with task‐specific motor practice [10, 39]. This pattern may indicate that tDCS‐related neuromuscular effects are more likely to emerge when stimulation is paired with exercises that directly recruit and overload the muscles subsequently evaluated by EMG.

Given that only two heterogeneous studies are available, the evidence is insufficient to draw firm conclusions regarding the effects of tDCS on muscle activation in individuals with ankle instability. More standardized and adequately powered trials with task‐specific EMG assessment are needed to clarify the conditions under which tDCS may influence neuromuscular function in this population.

### 4.4 Mechanistic Analysis

Several plausible neurophysiological mechanisms may explain why tDCS enhanced the effects of rehabilitation interventions in some trials but not others. At the cortical level, anodal tDCS is thought to increase neuronal excitability and promote LTP‐like plasticity in task‐relevant motor representations, thereby facilitating motor learning when paired with practice [34, 35, 36]. Depending on the cortical target and behavioral task, these neuroplastic effects may influence balance control, dynamic joint mechanics, or muscle activation through partially distinct sensorimotor pathways. Importantly, all included studies primarily employed anodal tDCS protocols intended to enhance cortical excitability, while the cathodal electrode mainly served as a return/reference electrode rather than an inhibitory stimulation target. Therefore, the potential differential effects of anodal versus cathodal stimulation paradigms on ankle instability outcomes remain unclear and warrant further investigation. When stimulation is delivered over M1 and paired with task‐specific motor practice (e.g., eccentric evertor strengthening, short‐foot/proprioceptive exercises, or sensorimotor‐focused rehabilitation approaches that directly activate peroneal or intrinsic foot muscles), the boosted corticomotor excitability may translate into measurable improvements in balance and movement kinematics [11, 12, 18, 28, 29]. By contrast, when stimulation targets are less focal (conventional montages with large return electrodes), when more focal stimulation approaches such as high‐definition tDCS are not used, or when stimulation is applied over regions less directly tied to the executed task (e.g., DLPFC), the induced modulation may be weaker or less functionally specific, producing null between‐group differences [25, 26, 27].

Other important factors include stimulation dose (current intensity and session number), timing relative to motor practice (concurrent or pre‐practice), and the specificity of the training task; mechanistic studies indicate that tDCS effects are most robust when stimulation coincides with repeated, task‐specific practice that engages the same cortical representations being modulated [42, 43]. This may partially explain why some studies using sensorimotor‐ or balance‐focused rehabilitation protocols reported additive effects of tDCS, whereas studies employing more reactive or agility‐based tasks demonstrated more variable findings. Inter‐individual factors, including baseline cortical excitability, age, sex, and the severity of sensorimotor impairment, together with the integrity of peripheral sensory input, may also modulate responsiveness to tDCS [44, 45]. Finally, high‐definition‐tDCS (more focal current delivery) may produce more targeted modulation of sensorimotor networks compared with conventional montages, potentially explaining why trials using high‐definition paradigms [11, 34] reported clearer additive effects on proprioception and balance. Taken together, these mechanistic considerations suggest that methodological heterogeneity, differences in rehabilitation protocols, and variability in stimulation parameters may have substantially contributed to the inconsistent findings observed across studies.

### 4.5 Clinical and Methodological Considerations

The present review demonstrated that tDCS delivered as an adjunct to rehabilitation interventions produced context‐dependent additive improvements in balance and ankle kinematics in several trials [11, 12, 18, 28, 29]. However, other trials found no significant between‐group differences for balance outcomes [25, 26, 27], and evidence for effects on muscle activation is inconsistent [27, 29]. A key driver of this heterogeneity appears to be methodological variation that interacts with underlying neurophysiology [34, 39]. In addition, one included study evaluated participants during the acute phase following ankle sprain [17], whereas the remaining studies focused on CAI. This population heterogeneity should be considered when interpreting the findings. Importantly, not all included interventions involved conventional exercise training paradigms. One study combined tDCS with joint mobilization techniques rather than active strengthening or balance exercises. Because mobilization‐based interventions may influence sensorimotor function through mechanisms distinct from active exercise training, direct comparison across studies should be interpreted with caution [12]. Another methodological limitation is that one included study did not employ sham stimulation, which increases the possibility of placebo and expectancy effects. Therefore, the findings of that study should be interpreted cautiously [12]. Finally, given the diversity of exercise modalities and therapeutic aims, the interventions were not considered mechanistically equivalent. This heterogeneity may have contributed to variability in treatment responses across studies.

Based on mechanistic literature and patterns observed across the included trials, we offer specific recommendations for future RCTs to reduce ambiguity and clarify clinical utility:

– Employ clearly defined stimulation parameters—including consistent current intensities, standardized session durations, and optimized electrode montages—so that interstudy comparisons become feasible. Dose–response studies would help determine the minimal effective exposure.
– Investigate whether targeting the M1, SMA, or alternative cortical regions yields differential effects on balance or ankle control.
– Examine the interaction between tDCS and specific exercise modalities, as the efficacy of tDCS may depend on the type, timing, and motor demands of the paired training.
– Future studies should also investigate the effects of tDCS during the acute phase following ankle sprain, as nearly all existing evidence is limited to CAI populations. Given the potential differences in sensorimotor impairment, cortical adaptation, and rehabilitation goals between acute and chronic phases, further research is needed to determine whether the effectiveness of tDCS may vary according to injury stage.
– Incorporate neurophysiological measures such as corticomotor excitability or sensorimotor mapping to clarify potential mechanisms.
– Evaluate long‐term retention to determine whether any observed improvements are sustained beyond the immediate post‐intervention period.

## 5 Conclusion

Synthesis of the available randomized trials suggests that tDCS, when applied as an adjunct to exercise‐based rehabilitation interventions, may provide additive improvements in balance and ankle kinematic measures in some contexts—particularly in CAI. However, findings are not uniform across studies, and evidence for changes in muscle activation is inconsistent. Therefore, the present literature does not support definitive clinical recommendations for routine use of tDCS in ankle instability; rather, it indicates potential, context‐dependent benefits on balance and kinematics that warrant confirmation in larger, methodologically standardized trials.

## Author Contributions

**Alireza Bayati:** investigation, methodology, writing – review and editing, writing – original draft. **Razieh Yousefian Molla:** project administration, writing – review and editing, writing – original draft, investigation, methodology, data curation, conceptualization.

All authors have read and approved the final version of the manuscript.

## Funding

The authors have nothing to report.

## Ethics Statement

The present study was a systematic review and did not include any human subjects. The review protocol was prospectively registered in the PROSPERO database (registration ID: CRD420251108595).

## Conflicts of Interest

The authors declare no conflicts of interest.

## Transparency Statement

Razieh Yousefian Molla affirms that this manuscript is an honest, accurate, and transparent account of the study being reported; that no important aspects of the study have been omitted; and that any discrepancies from the study as planned have been explained.

## Supporting information


Supporting File

## Data Availability

Data sharing is not applicable to this article as no datasets were generated or analyzed during the current study. Razieh Yousefian Molla had full access to all data in this study and takes complete responsibility for the integrity of the data and the accuracy of the data analysis.
