# Supplementary material for: The Additive Effects of Transcranial Direct Current Stimulation Combined With Exercise‐Based Rehabilitation Interventions on Balance, Ankle Kinematics, and Muscle Activity in Individuals With Ankle Instability: A Systematic Review
Source: Health Sci Rep. 2026 Jul 20;9(7):e72833. doi: 10.1002/hsr2.72833 (PMC13386100; doi:10.1002/hsr2.72833)
Supplement: Supplementary file 1 — Supporting File [file HSR2-9-e72833-s001.docx]

**Supplemental Appendix**

**Search strategy for PubMed: 109**

(((((((Transcranial Electrical Stimulation) OR (non-invasive brain stimulation)) OR (Transcranial direct current stimulation)) OR (Transcranial current stimulation)) OR (tDCS)) OR (neuromodulation)) AND (((((((ankle sprain) OR (ankle instability)) OR (ankle inversion)) OR (syndesmotic injury)) OR (syndesmotic injuries)) OR (ankle injury)) OR (ankle injuries))) AND ((((((((((((balance) OR (equilibrium)) OR (postural sway)) OR (postural stability)) OR (center of pressure)) OR (kinematic)) OR (walking)) OR (gait)) OR (standing)) OR (muscle activity)) OR (muscle activation)) OR (electromyography))

**Search strategy for Scopus: 281 (article: 276, conference papre: 5)**

(ALL(“Transcranial Electrical Stimulation”) OR ALL (“non-invasive brain stimulation”) OR ALL (“Transcranial direct current stimulation”) OR ALL(“Transcranial current stimulation”) OR ALL(tDCS) OR ALL(neuromodulation)) AND (ALL(“ankle sprain”) OR ALL(“ankle instability”) OR ALL(“ankle inversion”) OR ALL(“syndesmotic injury”) OR ALL(“syndesmotic injuries”) OR ALL(“ankle injury”) OR ALL (“ankle injuries”)) AND (ALL(balance) OR ALL(equilibrium) OR ALL(“postural sway”) OR ALL(“postural stability”) OR ALL(“center of pressure”) OR ALL(kinematic) OR ALL(walking) OR ALL(gait) OR ALL(standing) OR ALL(“muscle activity”) OR ALL(muscle activation) OR ALL(electromyography))

**Search strategy for Web of Science: 85 (article: 85, conference papre: 0)**

(((((ALL=(Transcranial Electrical Stimulation)) OR ALL=(non-invasive brain stimulation)) OR ALL=(Transcranial direct current stimulation)) OR ALL=(Transcranial current stimulation)) OR ALL=(tDCS)) OR ALL=(neuromodulation) AND ((((((ALL=(ankle sprain)) OR ALL=(ankle instability)) OR ALL=(ankle inversion)) OR ALL=(syndesmotic injury)) OR ALL=(syndesmotic injuries)) OR ALL=(ankle injury)) OR ALL=(ankle injuries) AND (((((((((((ALL=(balance)) OR ALL=(equilibrium)) OR ALL=(postural sway)) OR ALL=(postural stability)) OR ALL=(center of pressure)) OR ALL=(kinematic)) OR ALL=(walking)) OR ALL=(gait)) OR ALL=(standing)) OR ALL=(muscle activity)) OR ALL=(muscle activation)) OR ALL=(electromyography)

**Search strategy for ProQuest: 95 (article: 93, theses: 2)**

(“Transcranial Electrical Stimulation” OR “non-invasive brain stimulation” OR “Transcranial direct current stimulation” OR “Transcranial current stimulation” OR tDCS OR neuromodulation) AND (“ankle sprain” OR “ankle instability” OR “ankle inversion” OR “syndesmotic injury” OR “syndesmotic injuries” OR “ankle injury” OR “ankle injuries”) AND (balance OR equilibrium OR “postural sway” OR “postural stability” OR “center of pressure” OR kinematic OR walking OR gait OR standing OR “muscle activity” OR “muscle activation” OR electromyography)

**Search strategy for Science Direct: 20**

(“Transcranial direct current stimulation” OR “Transcranial current stimulation”) AND (“ankle sprain” OR “ankle instability” OR “ankle injury”) AND (balance OR kinematic OR electromyography)

**Search strategy for Cochrane Library: 30**

(“Transcranial Electrical Stimulation” OR “non-invasive brain stimulation” OR “Transcranial direct current stimulation” OR “Transcranial current stimulation” OR tDCS OR neuromodulation) AND (“ankle sprain” OR “ankle instability” OR “ankle inversion” OR “syndesmotic injury” OR “syndesmotic injuries” OR “ankle injury” OR “ankle injuries”) AND (balance OR equilibrium OR “postural sway” OR “postural stability” OR “center of pressure” OR kinematic OR walking OR gait OR standing OR “muscle activity” OR “muscle activation” OR electromyography)
